# Supplementary material for: Explaining Conformational Diversity in Protein Families through Molecular Motions
Source: Sci Data. 2024 Jul 10;11:752. doi: 10.1038/s41597-024-03524-5 (PMC11237097; doi:10.1038/s41597-024-03524-5)
Supplement: Supplementary file 1 — Supplementary Information [file 41597_2024_3524_MOESM1_ESM.pdf]

Supplemental Material for Explaining Conformational Diversity in Protein Families through Molecular Motions

Valentin Lombard[1], **Sergei Grudinin**[*2], **Elodie Laine**[*1,3]

[1] Sorbonne Université, CNRS, IBPS, Laboratoire de Biologie Computationnelle et Quantitative (LCQB), 75005 Paris, France.
[2] Université Grenoble Alpes, CNRS, Grenoble INP, LJK, 38000 Grenoble, France.
[3] Institut universitaire de France (IUF).

[*] corresponding authors: sergei.grudinin@univ-grenoble-alpes.fr, elodie.laine@sorbonne-universite.fr

Supplemental tables and figures

Supplemental Table S1: **Execution time on the PDB (748 297 protein chains)**

**Step**	**Library or tool**	**(CPU)**	**Time#**
a- Sequence extraction	GEMMI	16	9min 30s
b- Sequence clustering	MMseqs2	16	15s
c- Sequence alignment	MAFFT	16	23min 54s
de- Structure extraction and alignment	GEMMI	16	88min 2s
f- Linear motion extraction	NumPy	16	11min 46s
**Total**			2h 14min

Supplemental Table S2: **Properties of the ensembles in the most conservative and the most relaxed set ups.**

Property	Id., Cov.	Min.	1st Quart.	Median	3rd Quart.	Max.	Mean ± Sd
Ensemble size	80,80	2.00	2.00	4.00	8.00	3 334	12.00 ± 45.80
(in conformation)	30,50	2.00	3.00	5.00	14.00	12694	24.64 ± 137.35
Reference length	80,80	5.00	112.00	207.00	335.00	4579.00	252.03 ± 223.35
(in residue)	30,50	5.00	81.00	165.00	304.00	4516.00	224.07 ± 231.74
Max deviation	80,80	0.00	0.52	1.10	2.45	72.93	2.39 ± 3.95
(in Å)	30,50	0.00	0.78	2.15	5.11	114.50	4.25 ± 5.88
Motion complexity[a]	80,80	1.00	2.00	3.00	4.00	118.00	3.89 ± 4.43
(in mode)	30,50	1.00	2.00	3.00	4.00	105.00	3.98 ± 4.45
1st mode contribution[a]	80,80	7.30	50.00	64.90	80.90	100	64.85 ± 19.99
(in percentage)	30,50	7.30	49.90	65.40	82.20	100	65.32 ± 20.31
1st mode collectivity[a]	80,80	0.30	13.30	30.20	50.90	98.20	33.07 ± 21.92
(in percentage)	30,50	0.30	15.90	29.40	48.90	98.20	33.26 ± 20.90

[a] To compute motion properties, we focused on the subset of ensembles with at least three members. Indeed, pairs of conformations trivially exhibit single-mode motions and are thus disregarded variability exhibited by pairs of conformations can be trivially explained by only one mode

Supplemental Table S3: **Properties of the ensembles chosen for benchmarking manifold learning techniques.**

**Reference PDB Id**	**Protein name**	**Size** (# conf.)	**Length** (# res.)	**Mean deviation** (Å)	**Max deviation** (Å)	**Motion complexity** (# mode)	**1st mode contrib.** (%)	**1st mode coll.** (%)
1AKEA	ADK	42	214	2.48 ± 2.78	7.30	1	0.963	0.462
2WJPA	MurD	22	435	2.58 ± 4.09	16.07	2	0.816	0.616
1IWOA	ATPase	104	994	7.12 ± 4.19	15.84	3	0.597	0.481
5KOYB	ABC	61	1181	5.95 ± 3.20	14.94	4	0.786	0.681
2KLRA	Crys	23	82	2.08 ± 1.24	5.37	6	0.502	0.311
1AH6A	HSP90	52	213	1.13 ± 1.10	4.56	7	0.410	0.105
1NIWA	CALM	388	136	10.38 ± 4.23	23.68	8	0.415	0.580
2G1TA	KIN	122	271	2.85 ± 1.88	8.89	9	0.669	0.086
6YXWC	RAS	744	167	1.56 ± 1.02	8.58	24	0.347	0.173
3J345	CAP	3334	231	4.06 ± 1.68	17.55	30	0.232	0.070

Supplemental Table S4: **Proportion of conformations reconstructed with high accuracy.**

Reference	Protein	PCA (in %)	Poly-kPCA (in %)	RBF-kPCA (in %)	Sigmoid-kPCA (in %)	UMAP (in %)
1AKEA	ADK	83	83	83	83	-
2WJPA	MurD	5	82	82	82	0
1IWOA	ATPase	0	0	0	0	0
5KOYB	ABC	18	28	30	18	0
2KLRA	Crys	96	96	96	91	83
1AH6A	HSP90	92	90	90	92	90
1NIWA	CALM	0	1	2	0	-
2G1TA	KIN	93	93	93	93	57
6YXWC	RAS	99	99	98	99	-
3J345	CAP	99	99	99	99	-

We consider reconstructions with RMSD errors lower than 2 Å as highly accurate. For the kPCA, we set the hyperparameters to the values leading to the lowest average error over each conformational collection (see **Supplementary Table S5**).

Supplemental Table S5: **Average reconstruction errors for unseen conformations and hyperparameter values.**

Protein name	Method	Kernel type	sigma	alpha	n_{neigh}	d_{min}	Mean RMSD (Å)
ADK	pca						1.635
ADK	kpca	rbf	5.96e+03	1.00E-14			1.633
ADK	kpca	poly	13.3	2.81e+03			1.598
ADK	kpca	sigmoid	0.309	32.4			1.525
MurD	pca						3.115
MurD	umap				2	0.223	3.928
MurD	kpca	rbf	0.494	1.00E+05			1.774
MurD	kpca	poly	33.9	2.81e+03			1.755
MurD	kpca	sigmoid	1.00E+05	1.00E+05			1.774
ATPase	pca						3.607
ATPase	umap				9	0.112	5.161
ATPase	kpca	rbf	569	1.46e-13			3.567
ATPase	kpca	poly	910	5.96e-14			3.591
ATPase	kpca	sigmoid	2.33e+03	5.96e-14			3.606
ABC	pca						2.599
ABC	umap				2	1	3.911
ABC	kpca	rbf	569	5.96e-14			2.554
ABC	kpca	poly	569	3.56e-13			2.533
ABC	kpca	sigmoid	3.73e+03	5.96e-14			2.600
Crys	pca						1.360
Crys	umap				2	0.001	1.676
Crys	kpca	rbf	86.9	1.26e-11			1.356
Crys	kpca	poly	112	5.18e-12			1.357
Crys	kpca	sigmoid	0.193	5.43			1.302
HSP90	pca						0.719
HSP90	umap				4	0.889	0.865
HSP90	kpca	rbf	54.3	7.54e-11			0.704
HSP90	kpca	poly	222	2.44e-14			0.711
HSP90	kpca	sigmoid	1.00E+03	1.00E-14			0.718
CALM	pca						4.075
CALM	kpca	rbf	356	1.46e-13			3.525
CALM	kpca	poly	356	8.69e-13			3.531
CALM	kpca	sigmoid	356	1.6e-08			4.060
KIN	pca						1.228
KIN	umap				11	0.001	1.871
KIN	kpca	rbf	222	7.54e-11			1.225
KIN	kpca	poly	222	4.5e-10			1.226
KIN	kpca	sigmoid	910	5.96e-14			1.228
RAS	pca						0.612
RAS	kpca	rbf	139	2.12e-12			0.605
RAS	kpca	poly	139	3.09e-11			0.606
RAS	kpca	sigmoid	910	2.12e-12			0.612
CAP	pca						1.014
CAP	kpca	rbf	222	8.69e-13			0.986
CAP	kpca	poly	222	1.26e-11			0.989
CAP	kpca	sigmoid	356	4.5e-10			1.014

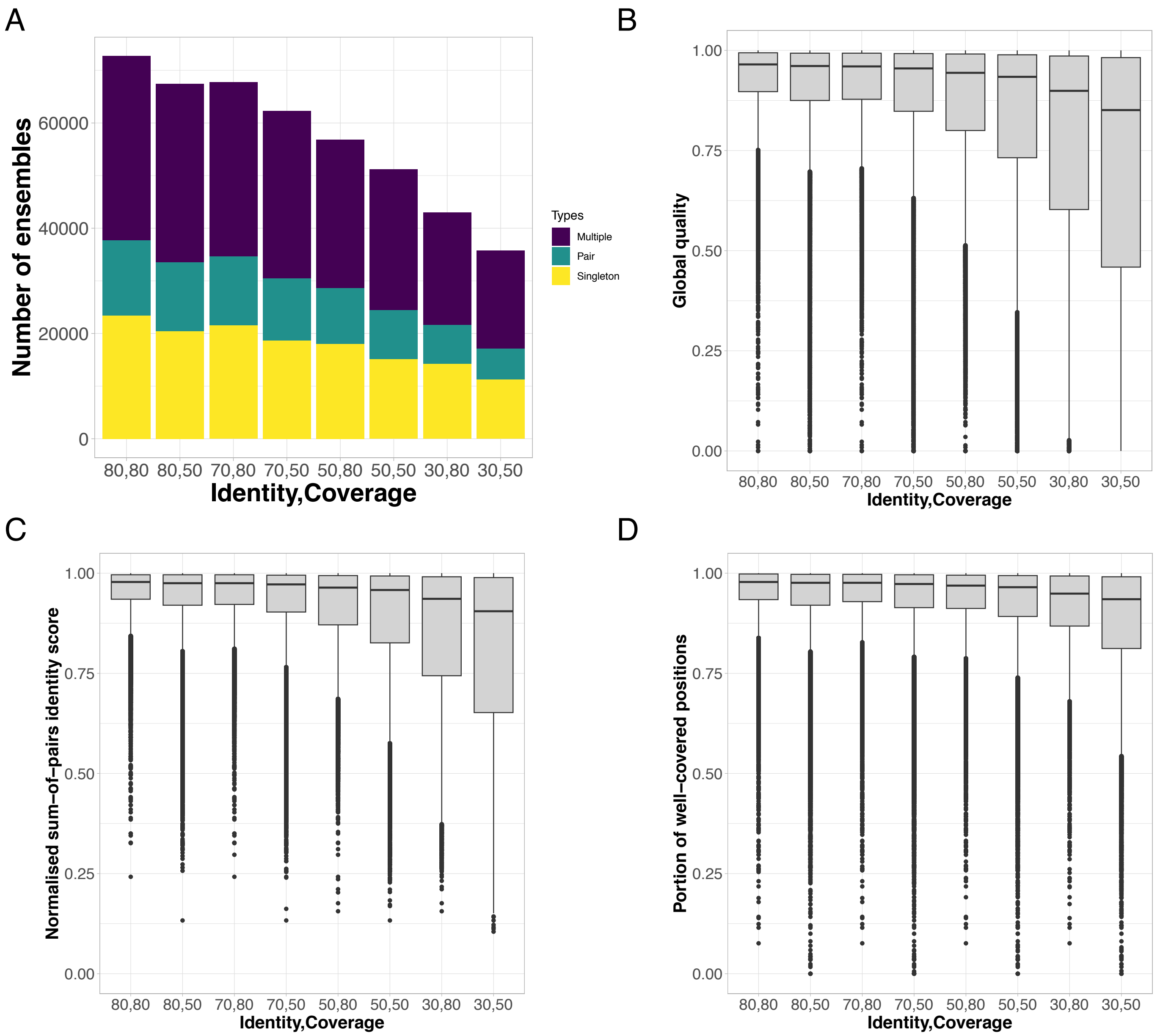

Supplemental Figure S1: **Global properties of the ensembles and their sequence alignments.** We report values computed across eight versions of the database, corresponding to eight combinations of sequence similarity and coverage thresholds. These combinations are given in x-axis. **A.** Number of singletons, pairs, and ensembles with at least 3 members. **B.** Distributions of sequence identity measured as a normalised sum-of-pairs scores with null mismatch and gap penalties. **C.** Distribution of coverage expressed as the fraction of positions with less than 80% gaps. **D.** Distribution of global alignment quality computed as a normalised sum-of-pairs scores with the following parameters: $\sigma_{match} = 1$, $\sigma_{mismatch} = \sigma_{gap} = -0.5$ (see *Methods*).

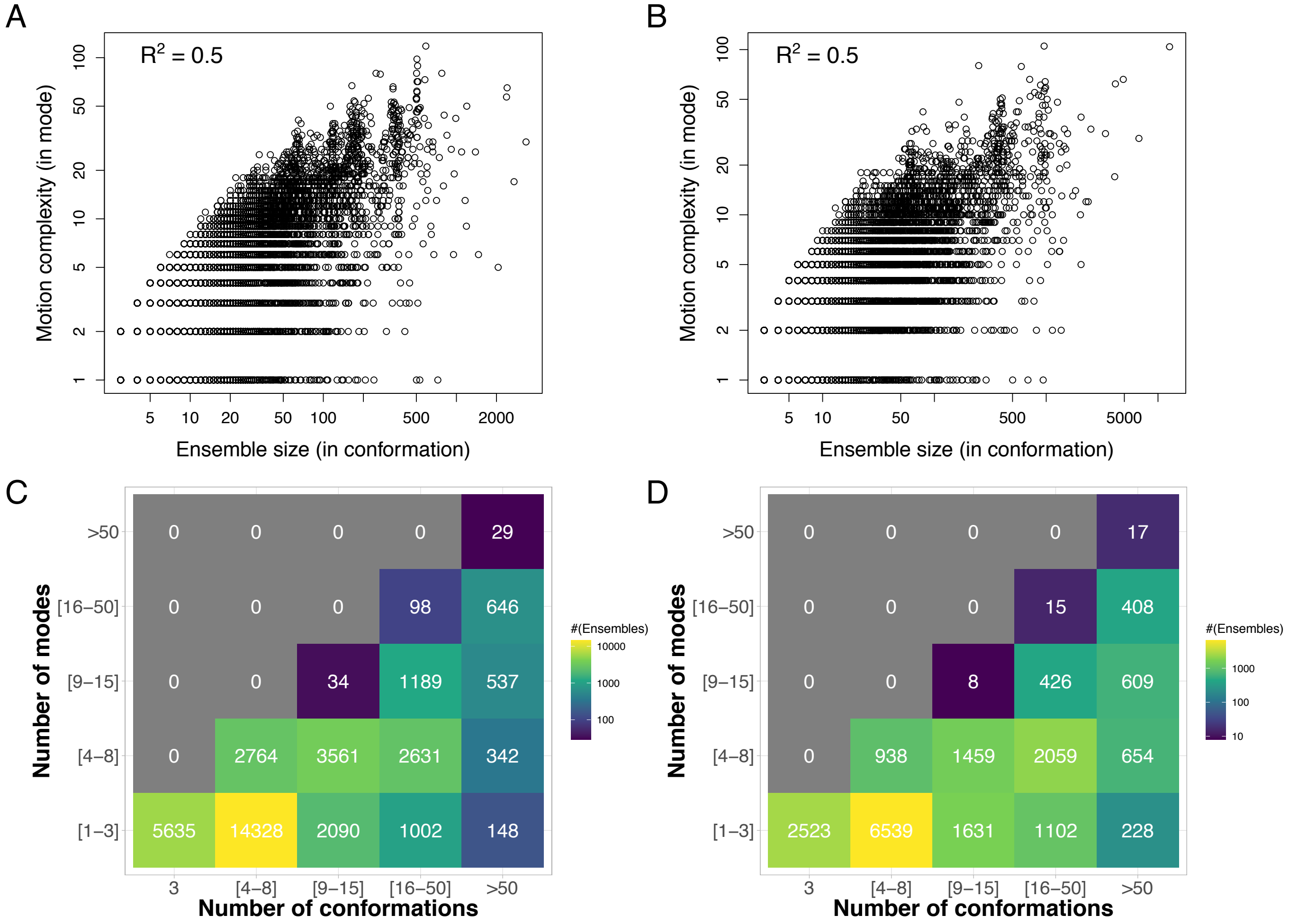

Supplemental Figure S2: **Influence of ensemble size on motion complexity** We report motion complexity, measured as the number of principal components or modes required to explain 80% of the positional variance, in function of the ensemble size, *i.e.* number of conformations. **A-B.** Scatterplots in log scale. **C-D.** Discretized heatmaps. We consider the most stringent set up, namely l_{80}^{80} (A,C), and the most relaxed one, namely l_{50}^{30} (B,D).

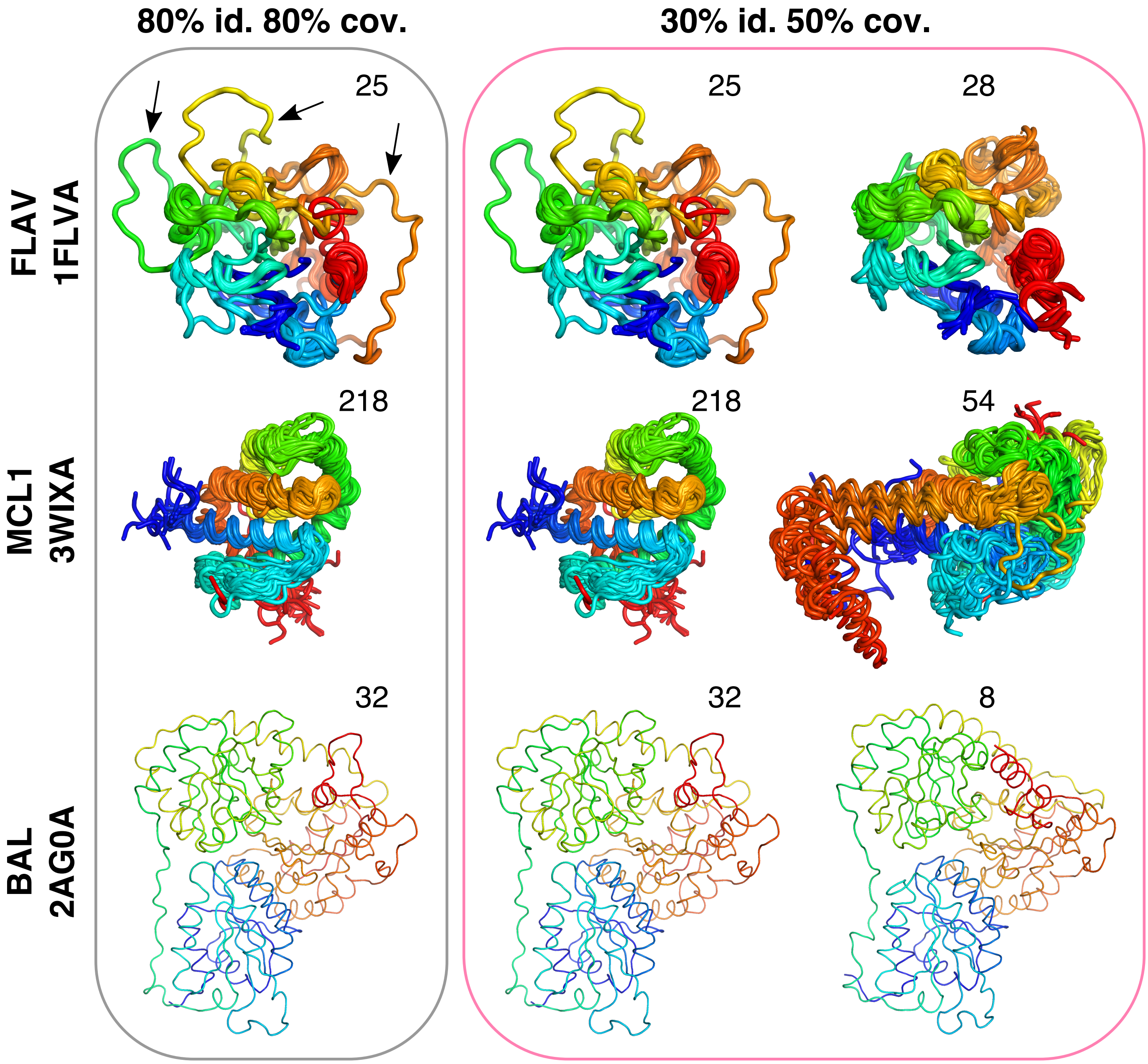

Supplemental Figure S3: **Expansion of three conformational ensembles upon relaxing sequence selection criteria.** We compare the set of conformations detected at two different levels of sequence similarity and coverage, namely l_{80}^{80} (on the left) and l_{50}^{30} (on the right). For the latter, we show separately the conformations already included in the ensemble at l_{80}^{80} (on the left) and the new additional conformations (on the right). The number of conformations in each (sub)ensemble is given on top. The color code indicates the position in the sequence, from the N-terminus in blue to the C-terminus in red. The flavodoxin (FLAV) ensemble contains one partially unfolded conformation, highlighted with the arrows. Some properties of these three examples are reported in Figure 2.

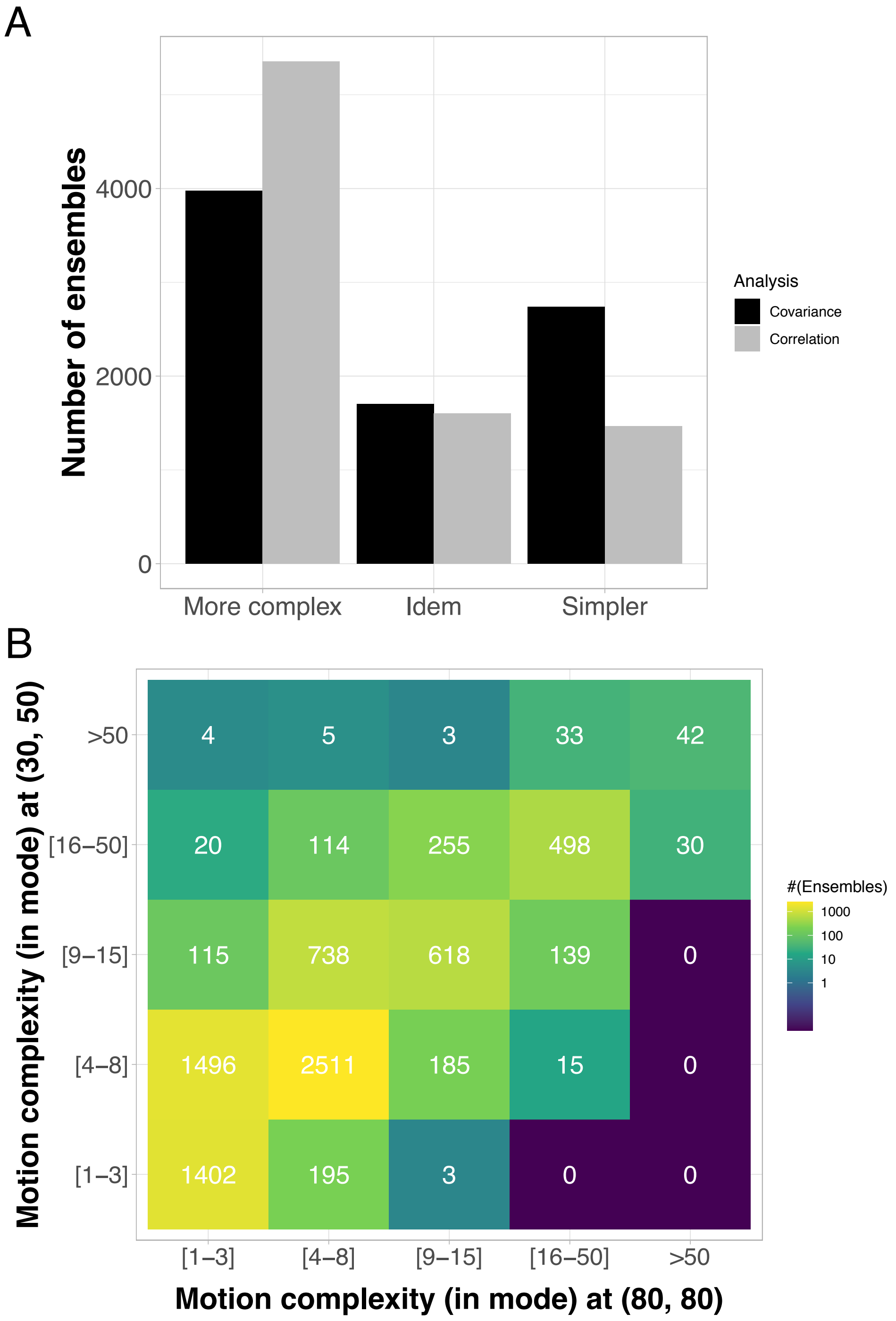

Supplemental Figure S4: **Evolution of motion complexity upon protein family expansion. A.** Number of ensembles where motion complexity increases, remains the same, or decreases between the most stringent and the most relaxed set ups. We extracted the motions from either the covariance (in black) or the correlation (in grey) matrix. **B.** Comparison of motion complexity estimated from the correlation matrix in the most stringent set up (x-axis) versus the most relaxed one (y-axis).

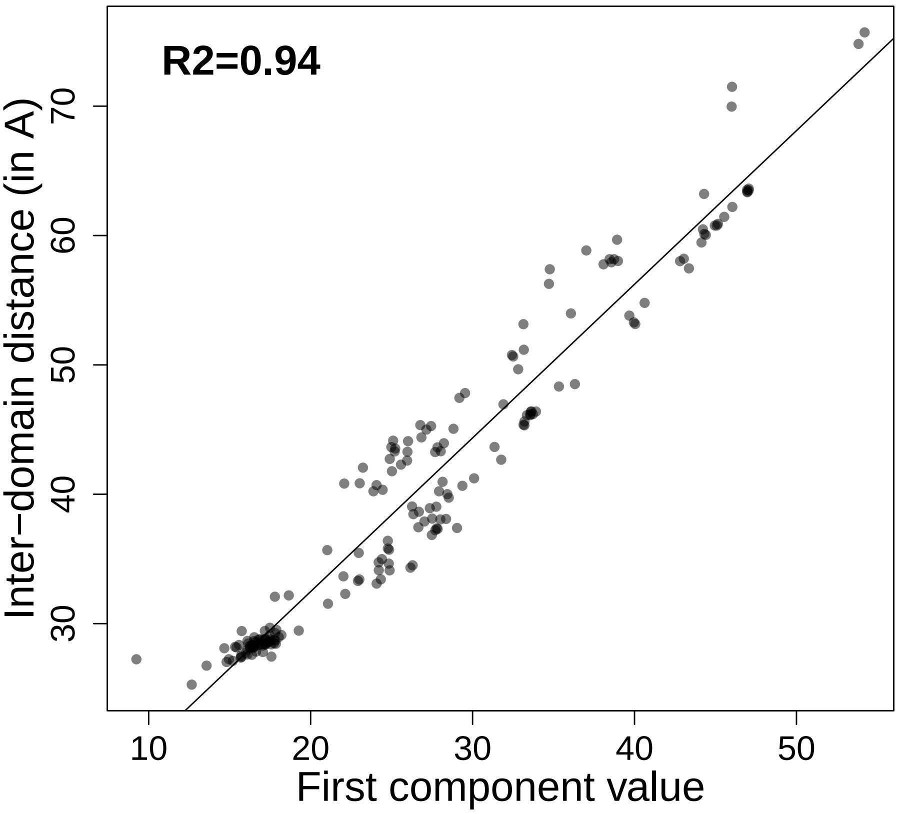

Supplemental Figure S5: **ABC protein opening in function of the first PCA component values.** The degree of opening of the ABC transporters is measured as the distance between the geometric centres of the two NBDs (in Å). The analysis is performed on the 188 conformations from the ABC structure similarity-based ensemble.

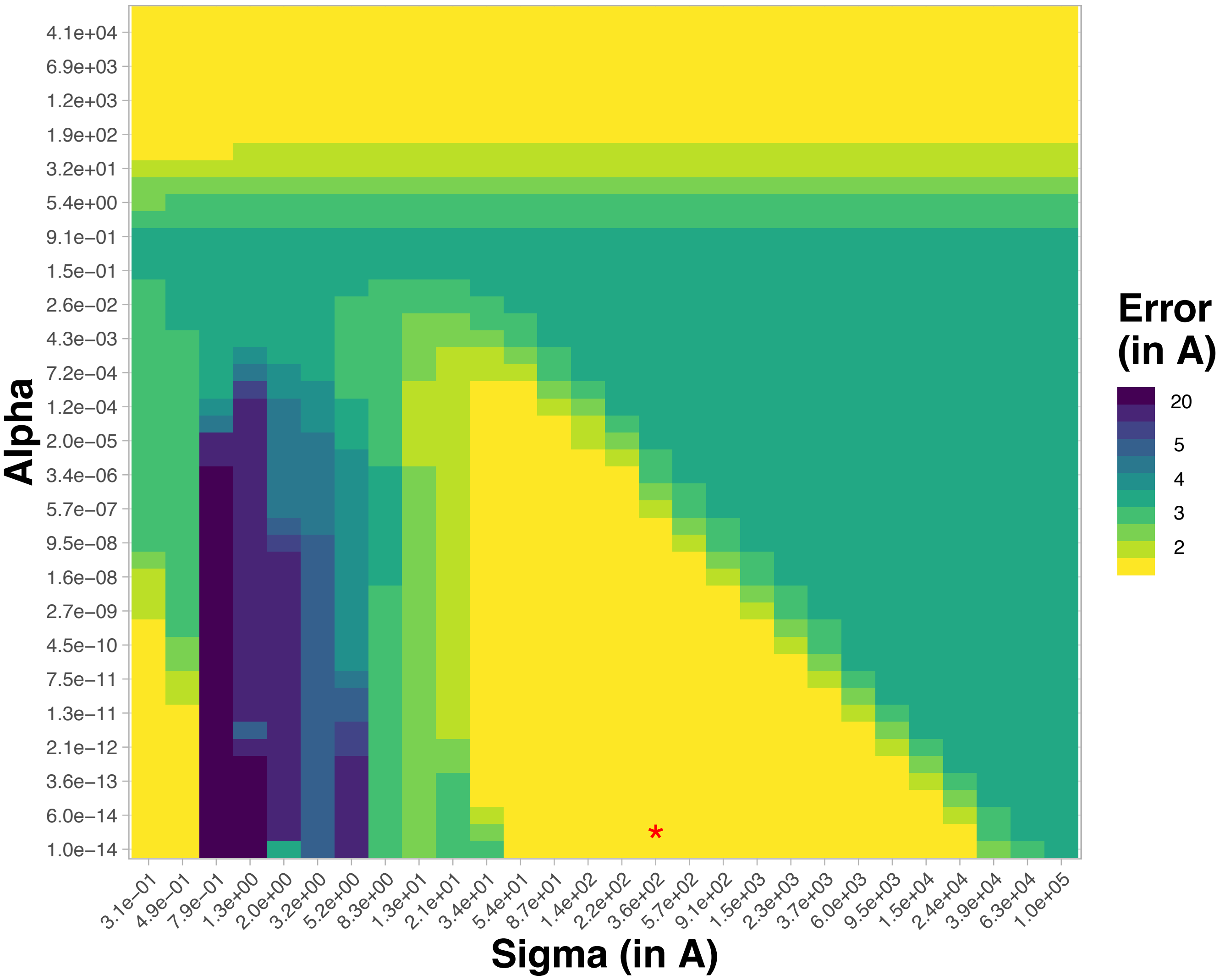

Supplemental Figure S6: **Systematic exploration of the two hyperparameters for kPCA-based conformation reconstruction.** We illustrate the influence of the hyper parameters σ and α on the reconstruction error (in Å) for a randomly picked up conformation (4th one) from the ADK protein ensemble. The red star highlights the optimal parameter values. We used the RBF kernel.

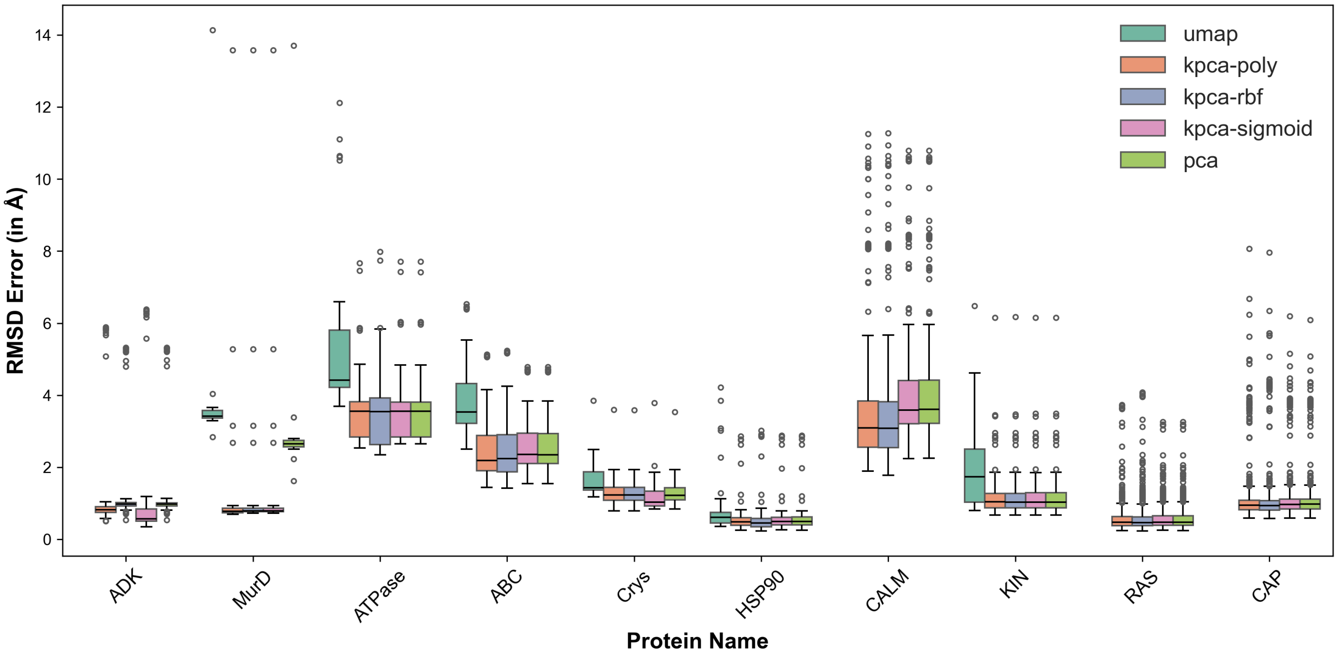

Supplemental Figure S7: **Distributions of the RMSD reconstruction errors (in Å) for each ensemble in the benchmark set.**We systematically reconstructed each conformation through a leave-one-cluster-out cross-validation procedure (see *Methods*). We set the hyperparameters of the kPCA and UMAP to the values yielding the best reconstruction, for each ensemble. The protein names in the x-axis are ordered according to motion complexity.

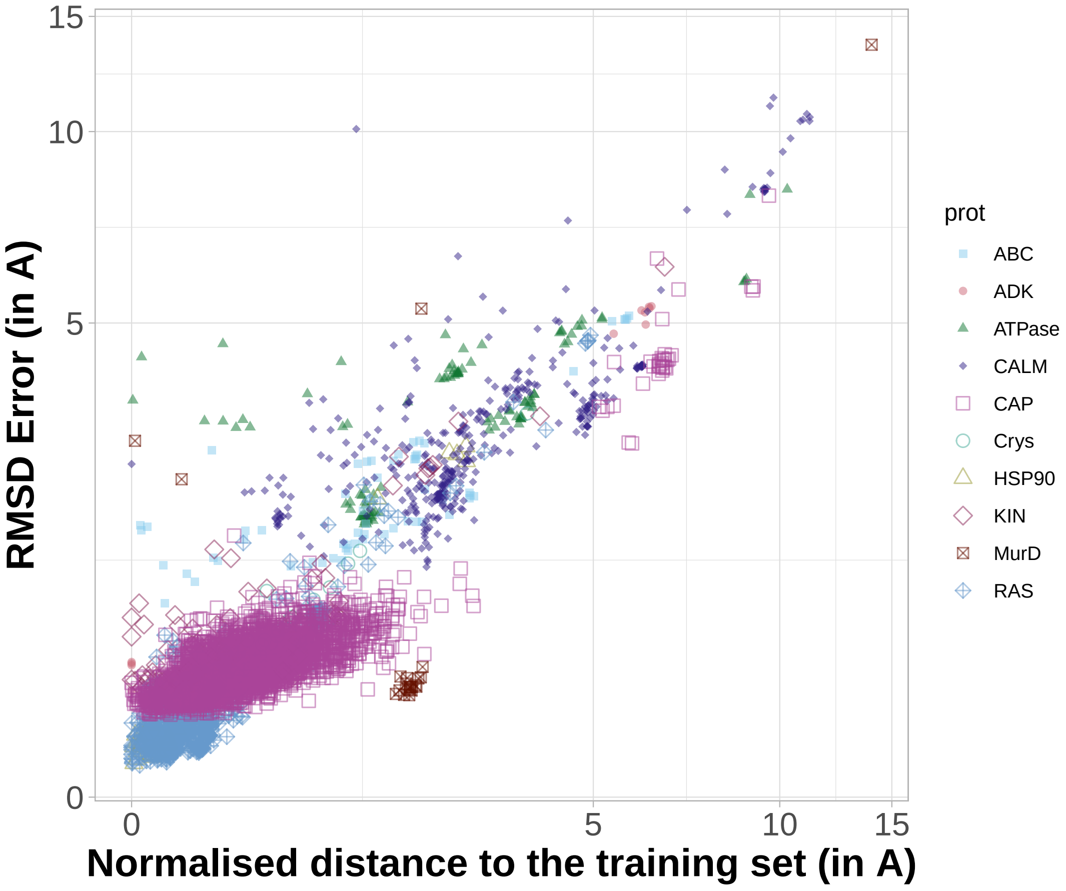

Supplemental Figure S8: **Reconstruction error in function of the distance to the training set for kPCA with RBF kernel.** The distance is computed between the test conformation and the convex hull defined by the training conformations in the low-dimensional representation space. It is normalised by the number of residues.

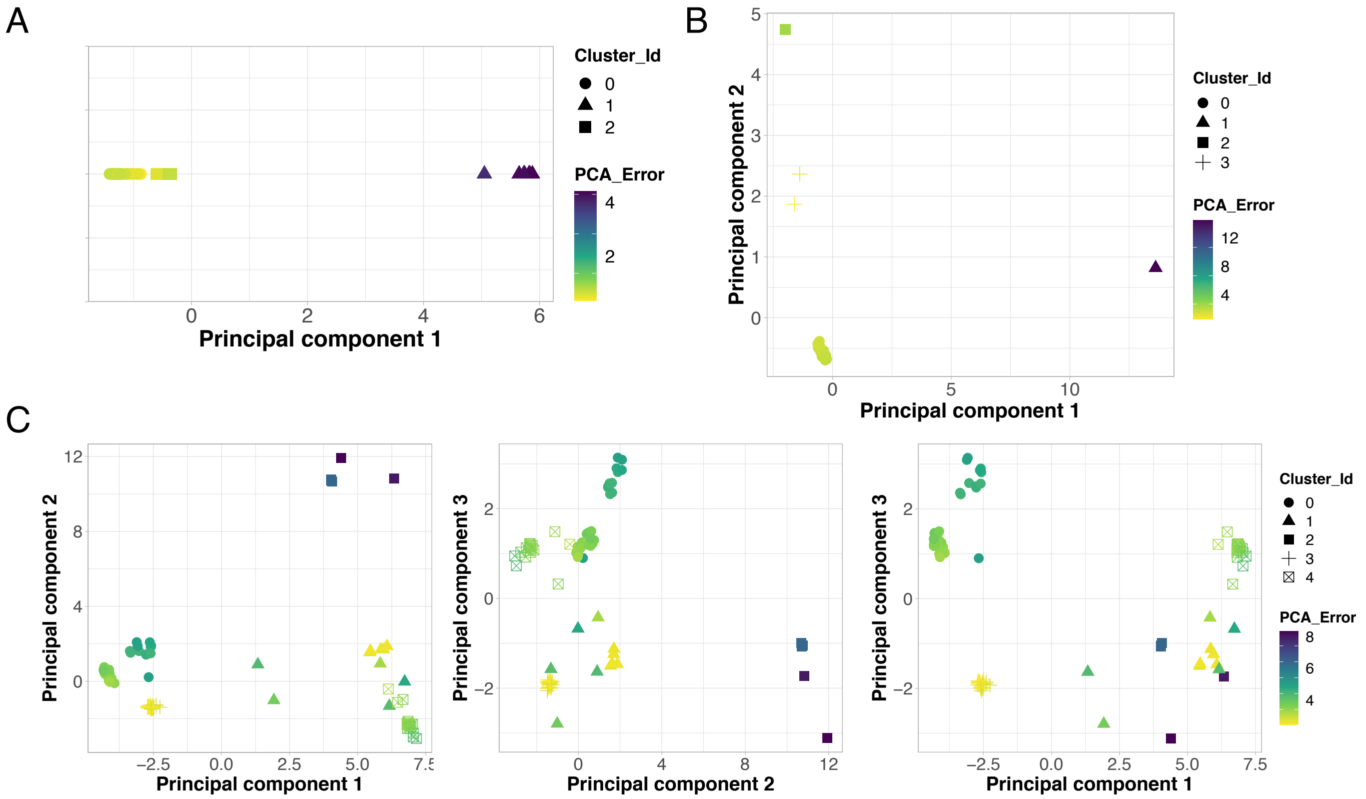

Supplemental Figure S9: **PCA feature spaces for three proteins from the benchmark.** We show the projections of the conformations in the l-dimensional PCA feature space, where l is the number of principal components needed to explain 90% of the total positional variance, for ADK (A), MurD (B) and ATPase (C). The point shapes indicate the clusters to which the conformations belong as determined by k-means clustering where $k = l + 2$. The colors reflect the RMSD reconstruction error (in Å). We reconstructed each conformation using the principal components computed from the set of conformations not belonging to the same cluster.

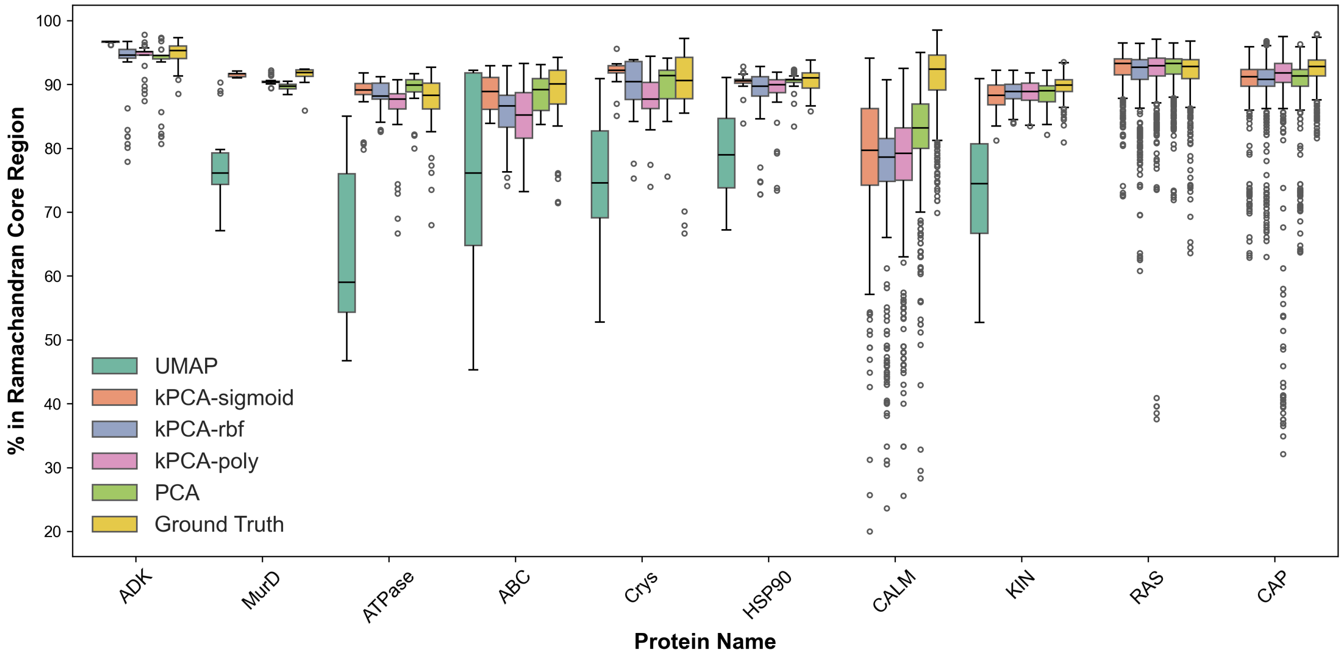

Supplemental Figure S10: **Distributions of the percentage of residues in the core region of the Ramachandran plot for each ensemble in the benchmark set.** We systematically reconstructed each conformation through a leave-one-cluster-out cross-validation procedure (see *Methods*). We set the hyperparameters of the kPCA and UMAP to the values yielding the best reconstruction, for each ensemble. The protein names on the x-axis are ordered according to motion complexity. The Ramachandran core region indicates the most favoured phi-psi angle combinations.

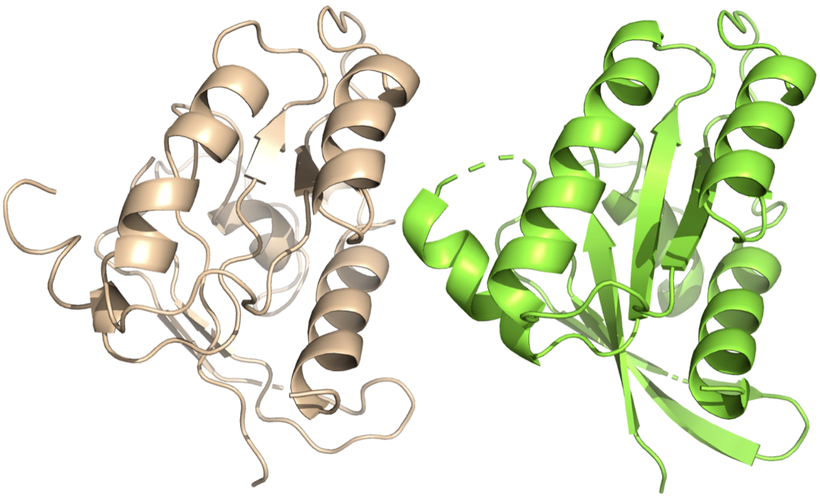

Supplemental Figure S11: **X-ray crystallographic structure of RAS (PDB code: 1PPL, chain A, in beige) and its PCA reconstruction (in green).** The PCA reconstruction displays a better secondary structure. The original structure has 63.9% of its residues in Ramachandran core region, while the PCA reconstruction has 96.4%.

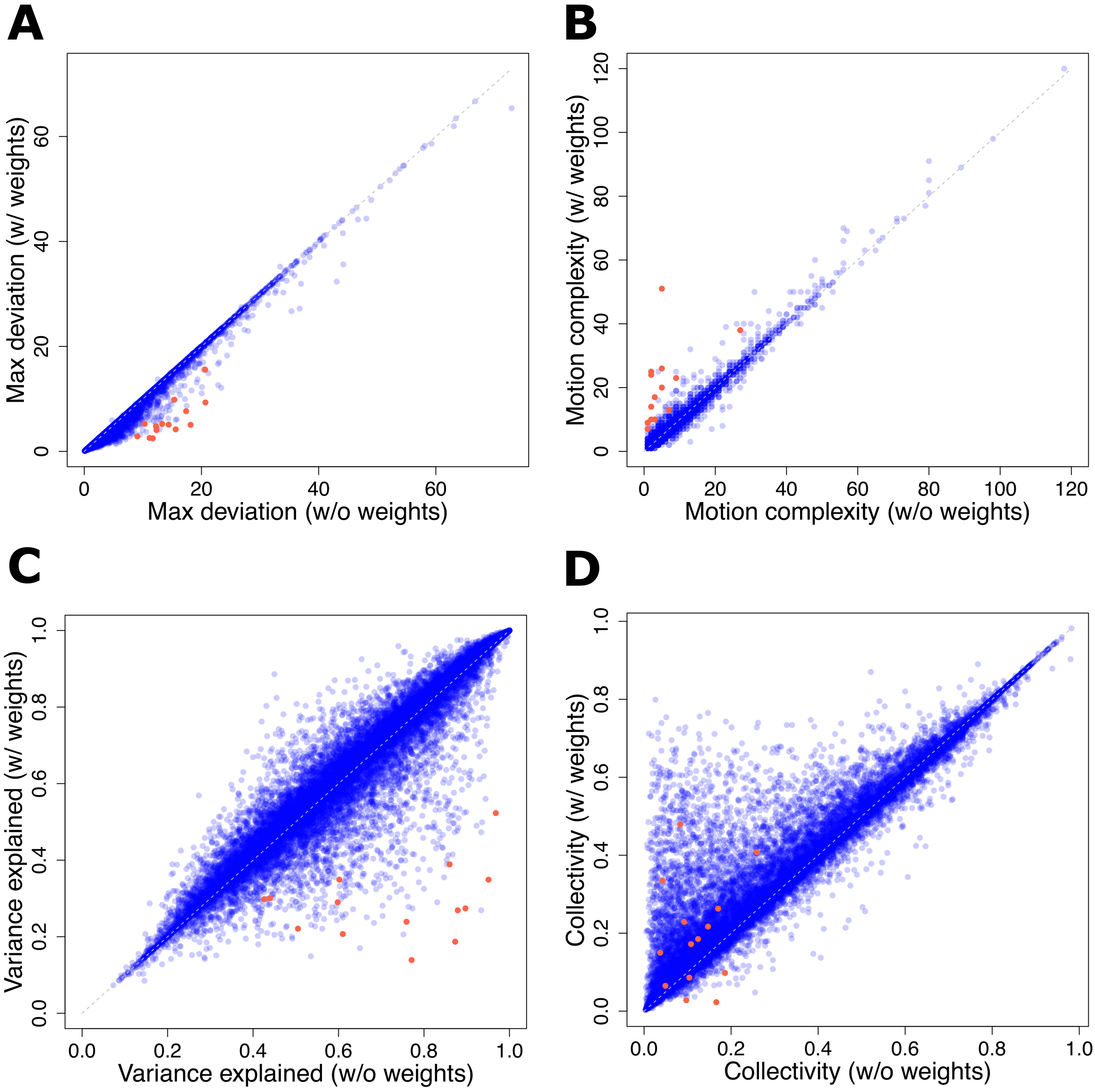

Supplemental Figure S12: **Influence of data uncertainty handling.** We compare some properties of the ensembles obtained at l_{80}^{80} when applying the weighting scheme accounting for uncertainty versus without weights. We consider only the ensembles with at least 3 members. **A.** Largest deviation between any two conformations (in Å). **B.** Motion complexity (in mode). **C.** Percentage of the variance explained by the most contributing linear motion. **D.** Collectivity of the most contributing linear motion. We highlight the ensembles for which applying the weighting scheme leads to a maximum deviation decrease of more than 5Å and an increased motion complexity by more than 5 modes in red.

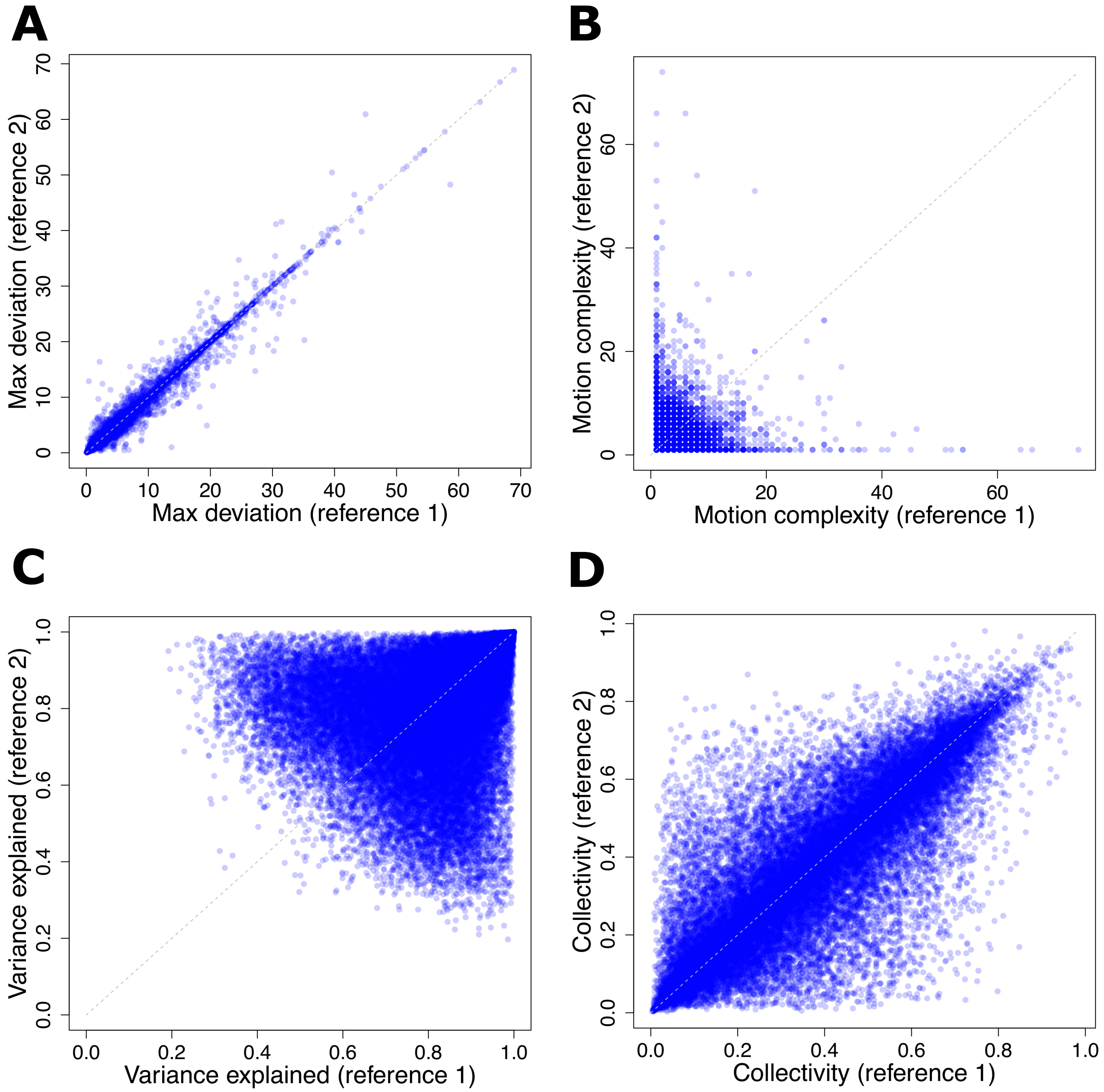

Supplemental Figure S13: **Influence of data conformation-specific centring.** We compare some properties of the ensembles obtained at l_{80}^{80} when superimposing and centring the conformations with respect to two different references. The first one is the closest to the multiple sequence alignment consensus (see *Materials and methods*). The second one has the highest RMS deviation from the first one. We consider only the ensembles with at least 3 members. **A.** Largest deviation between any two conformations (in Å). **B.** Motion complexity (in mode). **C.** Percentage of the variance explained by the most contributing linear motion. **D.** Collectivity of the most contributing linear motion.

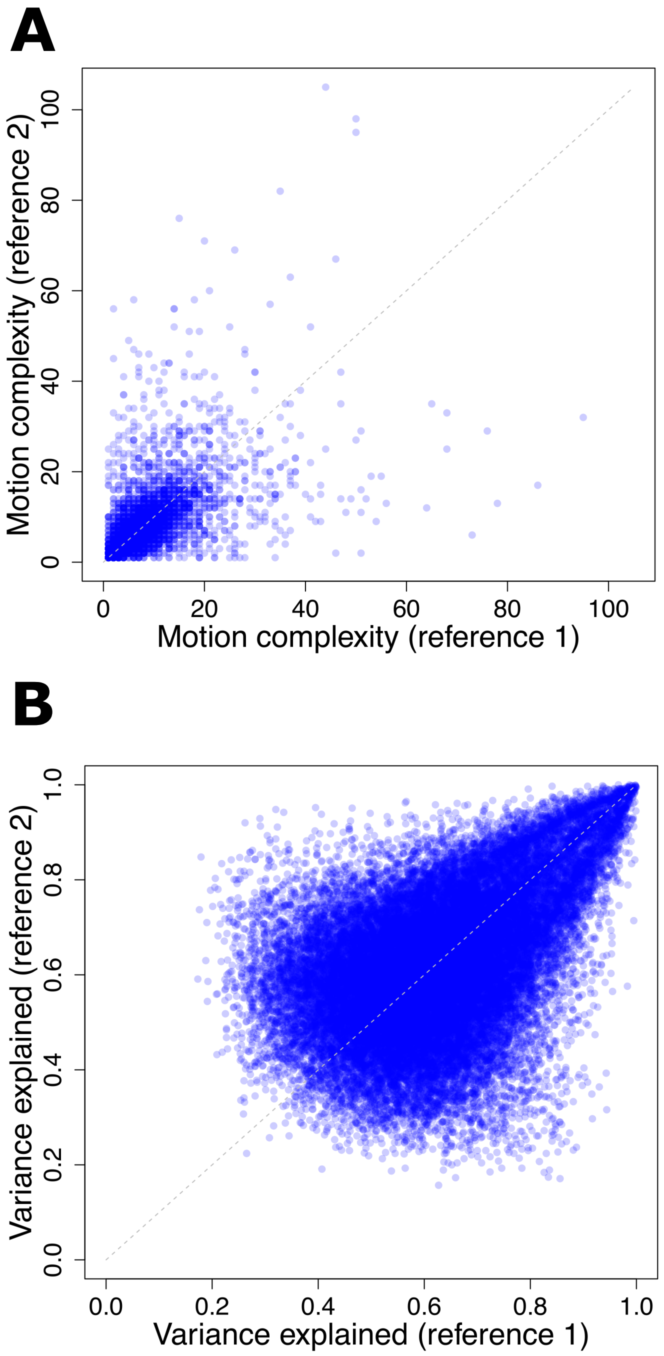

Supplemental Figure S14: **Influence of data conformation-specific centring when extracting motions from the correlation matrix.** We consider the ensembles with at least 3 members produced at l_{80}^{80} when superimposing and centring the conformations with respect to two different references. The first one is the closest to the multiple sequence alignment consensus (see *Materials and methods*). The second one has the highest RMS deviation from the first one. **A.** Motion complexity (in mode). **B.** Percentage of the variance explained by the most contributing linear motion. These plots can be compared with panels B and C from Figure S9.
